# Access to primary healthcare services and associated factors in urban slums in Nairobi-Kenya

**DOI:** 10.1186/s12889-020-09106-5

**Published:** 2020-06-22

**Authors:** Peter O. Otieno, Elvis O. A. Wambiya, Shukri M. Mohamed, Martin Kavao Mutua, Peter M. Kibe, Bonventure Mwangi, Hermann Pythagore Pierre Donfouet

**Affiliations:** African Population and Health Research Center, APHRC Campus, 2nd Floor, Manga Close, Off Kirawa Road, P.O. Box: 10787-00100, Nairobi, Kenya

**Keywords:** Access to primary healthcare, Universal health coverage, Urban slums, Penchansky and Thomas’s model

## Abstract

**Background:**

Access to primary healthcare is crucial for the delivery of Kenya’s universal health coverage policy. However, disparities in healthcare have proved to be the biggest challenge for implementing primary care in poor-urban resource settings. In this study, we assessed the level of access to primary healthcare services and associated factors in urban slums in Nairobi-Kenya.

**Methods:**

The data were drawn from the Lown scholars’ study of 300 randomly selected households in Viwandani slums (Nairobi, Kenya), between June and July 2018. Access to primary care was measured using Penchansky and Thomas’ model. Access index was constructed using principal component analysis and recorded into tertiles with categories labeled as poor, moderate, and highest. Generalized ordinal logistic regression analysis was used to determine the factors associated with access to primary care. The adjusted odds ratios (AOR) and 95% confidence intervals were used to interpret the strength of associations.

**Results:**

The odds of being in the highest access tertile versus the combined categories of lowest and moderate access tertile were three times higher for males than female-headed households (AOR 3.05 [95% CI 1.47–6.37]; *p* < .05). Households with an average quarterly out-of-pocket healthcare expenditure of ≥USD 30 had significantly lower odds of being in the highest versus combined categories of lowest and moderate access tertile compared to those spending ≤ USD 5 (AOR 0.36 [95% CI 0.18–0.74]; *p* < .05). Households that sought primary care from private facilities had significantly higher odds of being in the highest versus combined categories of lowest and moderate access tertiles compared to those who sought care from public facilities (AOR 6.64 [95% CI 3.67–12.01]; *p* < .001).

**Conclusion:**

In Nairobi slums in Kenya, living in a female-headed household, seeking care from a public facility, and paying out-of-pocket for healthcare are significantly associated with low access to primary care. Therefore, the design of the UHC program in this setting should prioritize quality improvement in public health facilities and focus on policies that encourage economic empowerment of female-headed households to improve access to primary healthcare.

## Background

Access to primary healthcare is widely acknowledged as key to reducing the global burden of morbidity and mortality [1–3]. In 1978, the World Health Organization launched the Alma Ata declaration in a bid to promote access to essential healthcare services while acknowledging health as “a foremost human right” [4]. While this agreement was ratified by a majority of low and middle-income (LMIC) countries, time has shown that the resolutions of the Alma Ata declaration remain unfulfilled globally [5]. In LMIC countries, access to primary care remains low [6]. Identifying the factors associated with access to primary healthcare in resource limited-settings is an essential undertaking, particularly, following the inclusion of universal health coverage (UHC) in the sustainable development goals [7]. In particular, access to primary healthcare services serves as an important proxy measure of UHC and thus can be used to evaluate the performance of a healthcare system and identify the untapped potential for advancing UHC [8].

Kenya typifies urban growth and slumization in sub-Saharan Africa. The majority (60–80%) of urban slum dwellers in Kenya live in informal settlements characterized by poor sanitation and hygiene, overcrowding and poor housing, and inadequate public healthcare services [9, 10]. Studies have also shown the burden of illness in urban informal settlements in Kenya tends to be high with poor coverage of effective preventive and therapeutic interventions [11–14]. The near absence of basic public amenities in slum settings in Kenya has resulted in the mushrooming of several small substandard clinics that are unable to offer integrated primary healthcare yet they serve a huge slum population [15]. This has negative implications on access to healthcare services among slum residents [16]. Even though the private health facilities have attempted to bridge the gap in demand for primary healthcare services in slum areas, previous studies have shown that they lack the capacity and do not guarantee service quality due to their profit-centric nature [17]. In spite of the recent efforts made by the Kenyan government to expand UHC, more evidence is needed on barriers and facilitators of access to healthcare services to improve coverage and performance of the primary healthcare system [18].

The objective of this study was to assess the factors associated with access to primary healthcare in urban slums in Nairobi, Kenya. Our study contributes to existing knowledge in two major ways. First, access to essential healthcare services is a critical indicator in the evaluation of UHC. Therefore, knowledge of healthcare services accessibility and associated factors in resource-poor urban settings may be used in the planning and delivery of evidence-based interventions to optimize health service delivery and achieve sustainability. Second, using Penchansky and Thomas’ model of access to healthcare [19], we consider a broader analysis of multiple indicators of access to primary healthcare ranging from health insurance coverage, timeliness of care, distance to the nearest primary care center, availability of essential healthcare services, affordability, acceptability to quality of care and treatment procedures.

## Methods

### Study design and setting

The data for this study are drawn from the Lown scholars’ survey on healthcare gaps in informal settlements conducted between June 2018 and July 2018 in Viwandani slums (Nairobi, Kenya). A total of 300 randomly selected households from the Nairobi Urban Health Demographic Surveillance System (NUHDSS) were surveyed in the Lown scholars’ study. The respondents comprised adults aged 18 years and over who were either household heads or permanent household members. The design of the Lown scholars’ study has been previously published [20].

### Data collection

A structured interviewer-administered questionnaire was used to collect data from the respondents. The interview questions consisted of healthcare utilization patterns, health insurance coverage, timeliness of care, distance to the nearest primary care center, availability of essential healthcare services, affordability, acceptability, quality of care, and treatment procedure. Other questions included perceived health status, out-of-pocket healthcare expenditure, and average monthly expenditure on health insurance. The responses were electronically recorded in a tablet. Secondary data on socioeconomic status of the selected households were obtained from the latest NUHDSS data, which is run by the African Population and Health Research Center in Viwandani and Korogocho slum settlements.

### Research model

Our study is underpinned by Penchansky and Thomas’ theory of access to primary healthcare [19]. Previous studies in Nigeria [21] and Istanbul [22] have also adopted this model. Penchansky and Thomas’ theory proposes a taxonomic definition of “access.” This theory summarizes a set of specific metrics that describe the fit between the healthcare system and the general population. These metrics are; availability, accessibility, accommodation, affordability, and acceptability of healthcare services. In particular, Penchansky and Thomas’ metrics of access form a formidable chain of access to primary care that is no stronger than its weakest link [19].

Using Penchansky and Thomas’ theory, we conceptualized seven independent and interconnected dimensions of access. These dimensions are health insurance coverage, timeliness of care, distance to the nearest primary care center, availability, affordability, and acceptability of essential healthcare services, quality of care and treatment procedure. Health insurance coverage measures the extent of financial protection of patients from the unexpected or high cost of healthcare services, distance to the nearest primary care center measures geographic accessibility of healthcare services. Timeliness of care measures the level of responsiveness of the health facility to the needs of the patient. Affordability measures the relationship between the costs of healthcare services versus the willingness and the ability of the patient to pay for the services. Availability determines the presence of requisite healthcare resources, such as infrastructure, personnel, technology, and essential supplies needed to meet the healthcare needs of the patients. Quality of care and treatment procedure reflects the operational organization of the provider in a manner that meets the preferences of the patients. Acceptability measures the extent of comfortability of the patients with immutable characteristics of the healthcare service provider such as sex, age, ethnicity, and social class.

### Measurements

#### Dependent variable

A proxy index for access to primary care was created based on healthcare utilization variables including health insurance coverage, timeliness of care, distance to the nearest primary care center, availability of essential healthcare services, affordability, acceptability, quality of care, and treatment procedure. The access index was finally computed using principal component analysis (PCA) and varimax rotation method. Principal components are weighted averages of the variables used to construct them. The computed index was finally used to classify the sampled households into three categories (tertiles): lowest, middle, and highest. The first eigenvalue of the PCA was 1.72 and the proportion of variance explained by the first three components was 58%.

#### Independent variables

We conceptualized two categories of predictor variables (individual and household-level factors) from Andersen and Newman’s behavioural model of health service utilization [23]. Individual factors comprised of age, marital status, and level of education. Household-level factors comprised of the sex of the household head, ethnicity, household size, wealth tertile, the primary source of care, and quarterly out-of-pocket (OOP) expenditure on healthcare. The quarterly OOP comprised of the total expenditures on consultation, diagnostics and laboratory tests, medication, emergency, and/or specialized care (such as dental care) in the three months preceding this survey. It was grouped into four categories: those who spent ≤ USD 5, between USD 5 and USD 9.9, between USD10 and USD 29.9 and ≥ USD 30. A wealth index was generated using PCA from the socio-economic variables including type of dwelling, ownership of the dwelling, construction materials of the dwelling, source of cooking fuel, the source of lighting fuel, household possessions/goods, the source of water for household consumption and type of sanitation facility. The households were grouped into tertiles based on the generated wealth index (lowest, middle, and highest).

#### Data analysis

The outcome variable for this study is access to primary healthcare services. Descriptive statistics were used to summarize the background characteristics of the respondents and the frequency distribution of access to primary care with individual and household-level factors. The outcome variable assumes an increasing order. Hence a multivariate ordered logistic regression was conducted. We fitted a proportional odds model; however, following the Brant test, we found that the critical assumption of parallel slopes^1^ was violated in some of the covariates (age group, education level, and primary source of care). Consequently, we implemented a partial proportional odds model which is less restrictive, and relaxes the proportional odds assumption, allowing the effect of the explanatory covariates to vary [24]. More information on the partial proportional odds model is available in numerous sources [24, 25]. All explanatory variables in the unadjusted partial proportional odds model that were associated with the outcome were added to the adjusted partial proportional odds model. The results of the multivariate logistic regression compare a continuum of households ranging from those that have low access, moderate access to those that have highest access to primary healthcare. We used Stata version 15.1 and statistical significance was defined as a *p*-value less than 0.05 (2-sided). The gologit2 [25] Stata command was used to fit the partial proportional odds model.

#### Ethical considerations

The protocol for this study was reviewed and approved by the African Medical and Research Foundation based in Nairobi, Kenya (*P482/2018*). Written informed consent was sought from all respondents prior to participation.

## Results

### Socio-demographic characteristics of the respondents

Table 1 shows the demographic characteristics of the respondents. Male respondents constituted a slightly higher proportion of the sample (52%) compared to females (48%). Approximately 44% of the respondents were aged between 30 and 44 years, 38% for 18–29 years, 14% for 45–59 years, and 3% for 60 years and above. The majority of the respondents (57%) were either married or living together with their partners. About 52% of the respondents had completed secondary education, 40% primary education, 7% tertiary education and 1.3% had no formal schooling. The majority of the respondents (59%) were Protestants, others included Catholics (32%), Muslims (3%), other Christians (2%), and, no religion (4%). Most of the households (61%) consisted of between one and three members. About 33% of the respondents were in the lowest, moderate, and highest wealth tertile respectively.

**Table 1:** Socio-demographic characteristics of the respondents

	Number	Percent
Sex		
Male	155	51.7
Female	145	48.3
Age group		
18–29	115	38.3
30–44	133	44.3
45–59	42	14.0
60 and above	10	3.3
Marital status		
Married/living together	171	57.0
Divorced/ separated	43	14.3
Widowed	8	2.7
Never married/never lived together	57	19.0
Others	21	7.0
Level of education		
No formal schooling	4	1.3
Primary	120	40.0
Secondary	156	52.0
College/university	20	6.7
Religion		
Roman catholic	96	32.0
Protestants	178	59.3
Other Christians.	6	2.0
Muslim	9	3.0
No religion	11	3.7
Household size		
1–3	183	61.0
4–6	104	34.0
7+	13	4.3
Wealth tertile		
Lowest	100	33.3
Middle	100	33.3
Highest	100	33.3
Health insurance coverage		
Yes	129	43.0
No	171	57.0

### Distribution of the respondents by access to primary care

Table 2 shows the distribution of respondents by access tertile. The age group 45 years and above had the highest percentage of respondents in the highest access tertile at 38.5%. A higher percentage of households headed by males (38.2%) were in the highest access tertile compared to those headed by females (16.4%). Respondents with primary level of education constituted the highest percentage of those in the highest access tertile at 36.3%. Households with 1–3 members had the highest proportion of those in the highest access tertile at 36%. Respondents whose main source of income is casual work constituted the highest percentage of those in the highest access tertile at 35.5%.

**Table 2:** Distribution of respondents by access to primary care

	N	Access tertiles		
		(%) lowest	(%) moderate	(%) highest
**Age group**				
18–29	115	33.91	28.70	37.39
30–44	133	37.59	34.59	27.82
45 and above	52	25.00	36.54	38.46
Sex of the household head**				
Male	233	29.18	32.62	38.20
Female	67	50.75	32.84	16.42
**Marital status**				
Married/living together	171	33.33	31.58	35.09
Divorced/separated/widowed	51	43.14	35.29	21.57
Never married	78	29.49	33.33	37.18
**Education**				
Primary and below	124	33.87	29.84	36.29
Secondary	156	34.62	32.69	32.69
Tertiary	20	30.00	50.00	20.00
**Household size**				
1–3	183	29.51	34.43	36.07
4 and above	117	41.03	29.91	29.06
**Occupation**				
Employed worker	65	38.46	26.15	35.38
Casual worker	141	25.53	39.01	35.46
Trader	73	39.73	32.88	27.40
Unemployed	21	57.14	9.52	33.33
**Wealth tertile**				
Lowest	100	30.00	35.00	35.00
Middle	100	39.00	33.00	28.00
Highest	100	33.00	30.00	37.00
Quarterly healthcare expenditure*				
≤ USD 5	143	22.38	39.16	38.46
USD 5- USD 999	42	45.24	23.81	30.95
USD10- USD 29.9	49	36.73	30.61	32.65
≥ USD 30	66	50.00	25.76	24.24
**Ethnicity**				
Kamba	107	28.97	31.78	39.25
Kikuyu	92	35.87	30.43	33.70
Kisii	20	50.00	30.00	20.00
Luhya	45	35.56	42.22	22.22
Luo	17	23.53	29.41	47.06
^1^Others	19	42.11	31.58	26.32
Source of primary care**				
Public	142	46.48	38.73	14.79
Private	158	22.78	27.22	50.00
**Total**	**300**	**34.00**	**32.67**	**33.33**

CI 95% confidence interval. *significant at *p* < 0.05, ** significant at *p* < 0.001, ^1^Other tribes comprise Borana, Taita, and Garre

Respondents in the highest wealth tertile constituted the highest percentage of those in the highest access tertile at 37%. Respondents whose average out-of-pocket healthcare expenditure in the three months preceding the survey was below USD 5 constituted the highest percentage of those in the highest access tertile at 39%. Respondents whose ethnic background is Luo constituted the highest percentage of those in the highest access tertile at 47%. A higher percentage of respondents who sought care from a private health facility (50%) were in the highest access tertile compared to those who sought care from a public health facility (14.8%).

### Factors associated with access to primary care

We first conducted the Brant test of the parallel lines assumption/proportional odds on the ordered logistic regression. The Brant test suggests that this hypothesis is violated (*χ*2 = 20.95, *p*-value = 0.051). Hence, we reported the results of the multivariate analysis using the generalized ordinal logistic regression in Table 3.

**Table 3:** Generalized ordinal logistic regression of factors associated with access to primary care

Access tertile		Unadjusted Model			Adjusted Model		
		OR	95% CI		OR	95% CI	
**Lowest**							
Sex of the household head (Ref: female)	Male	2.50*	1.43	4.36	2.07*	1.14	3.76
Quarterly healthcare expenditure	USD 5- USD 999	0.35*	0.17	0.72	0.27*	0.13	0.59
(Ref:≤USD 5)	USD 10- USD 29.9	0.50	0.25	1.00	0.39*	0.18	0.83
	≥USD 30	0.29**	0.15	0.54	0.24**	0.12	0.47
Source of primary care (Ref: public)	Private	2.94**	1.79	4.84	3.56**	2.07	6.11
Age group (Ref: 18–29)	30–44	0.85	0.51	1.44			
	45–59	1.28	0.59	2.78			
	> 60	4.62	0.56	37.78			
Education (Ref: primary)	Secondary	0.97	0.59	1.59			
	Tertiary	1.20	0.43	3.33			
Household size (Ref: 1 to 3)	4+	0.60	0.37	0.98			
Marital status (Ref: married/living together)	Single/divorced/separated/widowed	0.93	0.58	1.51			
Wealth tertile (Ref: lowest)	Middle	0.67	0.37	1.21			
	Highest	0.87	0.48	1.58			
Ethnicity (Ref: Kamba)	Kikuyu	0.73	0.40	1.32			
	Kisii	0.41	0.15	1.08			
	Luhya	0.74	0.35	1.55			
	Luo	1.33	0.40	4.38			
	Others	0.56	0.21	1.53			
**Moderate**							
Sex of the household head (Ref: female)	Male	2.50*	1.43	4.36	3.05*	1.47	6.37
Quarterly healthcare expenditure	USD 5- USD 999	0.72	0.34	1.50	0.51	0.23	1.13
(Ref:≤USD 5)	USD 10- USD 29.9	0.78	0.39	1.54	0.57	0.27	1.21
	≥USD 30	0.51*	0.27	0.99	0.36*	0.18	0.74
Source of primary care (Ref: public)	Private	5.76**	3.30	10.07	6.64**	3.67	12.01
Age group (Ref: 18–29)	30–44	0.65	0.38	1.10			
	45–59	1.26	0.61	2.58			
	> 60	0.42	0.08	2.06			
Education (Ref: primary)	Secondary	0.85	0.52	1.40			
	Tertiary	0.44	0.14	1.39			
Household size (Ref: 1 to 3)	4+	0.73	0.44	1.20			
Marital status (Ref: married/living together)	Divorced/separated/widowed	0.83	0.51	1.35			
Wealth tertile (Ref: lowest)	Middle	0.72	0.40	1.32			
	Highest	1.09	0.61	1.94			
Ethnicity (Ref: Kamba)	Kikuyu	0.73	0.40	1.32			
	Kisii	0.41	0.15	1.08			
	Luhya	0.74	0.35	1.55			
	Luo	1.33	0.40	4.38			
	Others	0.56	0.21	1.53			

*CI* confidence interval, *Ref* reference category, *significant at *p < 0.05, *** significant at *p* < 0.001

The odds of being the combined categories of moderate and highest access tertiles versus lowest were about twice higher for male-headed households than female-headed households, other variables held constant (AOR 2.07 [95% CI 1.14–3.76]; *p* < .05). In the same vein, the odds of being in the highest access tertile versus the combined categories of lowest and moderate access tertile were three times higher for male than female-headed households, given the other variables are held constant (AOR 3.05 [95% CI 1.47–6.37]; *p* < .05).

The odds of being in the combined moderate and highest access tertiles versus lowest were 76% lower for households with an average quarterly out-of-pocket healthcare expenditure of USD 30 and above compared to those with a quarterly expenditure of less than USD 5, given that other variables are held constant (AOR 0.24 [95% CI 0.12–0.47]; *p* < .001). Likewise, the odds of being in the highest versus combined categories of lowest and moderate access tertile were 64% lower for households with an average quarterly out-of-pocket healthcare expenditure of USD 30 and above compared to those with a quarterly expenditure of less than USD 5, given the other variables are held constant (AOR 0.36 [95% CI 0.18–0.74]; *p* < .05).

The odds of being in the combined categories of moderate and highest access tertiles versus lowest access tertile were about four times higher for households that sought care from private facilities compared to the public, given the other variables are held constant (AOR 3.56 [95% CI 2.07–6.11]; *p* < .001). In the same vein, the odds of being in the combined categories of lowest and moderate access tertile versus highest access tertile were about seven times higher for households that sought care from private facilities compared to public, given the other variables are held constant (AOR 6.64 [95% CI 3.67–12.01]; *p* < .001).

## Discussion

In this study, we assessed the level of access to primary healthcare services and associated factors in urban slums in Nairobi-Kenya. Our findings revealed that seeking care from a public primary care facility, living in a household headed by a female, and paying out-of-pocket for healthcare services are significantly associated with low access to primary care.

Our results suggest a systematic disadvantage of female versus male-headed households in access to primary care. Previous studies have also consistently revealed widespread gender differences in access to primary care [26–28]. Several explanations have been offered. These disparities may be associated with gender inequalities in access to health insurance coverage [29, 30]. Our findings are consistent with the results of the 2013 Kenya Household Healthcare Expenditure and Utilization Survey (KHEUS) which suggests that women are more likely to have poor access to primary healthcare services due to low health insurance coverage [31]. In general, the most important determinants of health insurance coverage are employment-related factors and income [16, 29, 32]. A possible explanation for these results could be the existence of gender differences in job opportunities with women more likely to engage in informal employment with a lack of entitlements such as health insurance than men [28].

Our finding that paying out-of-pocket for healthcare services was significantly associated with low access to primary care is consistent with evidence from a number of recent studies [33–36]. In particular, poor financial protection in low resource settings is attributable to inadequate funding, fragmentation of healthcare resources, and inadequate health insurance coverage [16]. Similar to the findings of our study, previous studies have also shown that direct OOP costs place a huge burden of bearing the costs of illness to the sick person and their households, and is therefore, a major contributor to inequities [37–41]. Providing financial risk protection to reduce OOP health expenditure as envisaged in the Sustainable Development Goals and UHC agendas are critical to improving access to primary healthcare in resource-poor urban settings.

Similar to previous studies [42–44], our results suggest higher odds of low access to primary care among households that sought primary healthcare services from public facilities compared to private. A plausible explanation for these findings could be due to in part the gaps in basic health infrastructure, medical equipment, availability of drugs, and other essential supplies in public primary care facilities [11, 39, 45, 46]. If not addressed, this lack of inputs may grossly affect the accessibility of primary services and jeopardize the efforts towards achieving UHC. The results of the current study findings reinforce the need for the Kenyan government to address the weaknesses of the public health sector and regulate the quality of public health facilities in urban slum settlements by providing technical support to bring quality healthcare services closer to slum populations.

Our findings have three key policy implications. First, within the healthcare system in Viwandani slums in Nairobi, Kenya, there is an urgent need for implementation of policies and programs that take into account the gender disparity in access to primary care. Second, there is a need to scale up efforts for expanding health insurance coverage in the slum communities to cushion households against OOP expenditures. Third, proper mechanisms should be developed to regularly monitor the quality of healthcare services offered by public facilities in urban slums by the relevant authorities. Technical support is also required for them to improve the quality of healthcare services.

### Limitations

The respondents for this study comprised adults aged 18 years and over who were either household heads or permanent household members, therefore, the possibility of recall bias or slight variations in the responses provided by the household heads, their spouses, or other credible adult household members cannot be overemphasized. The study did not disaggregate health expenditure into the various components of direct and indirect healthcare spending. There is therefore little room to draw conclusive arguments on costs such as transport to access healthcare, time lost from work and other informal costs which households may incur in the process of seeking access to care. In addition, the fact that the study was conducted in one urban slum settlement may have a bearing on its generalizability to other urban slums in Kenya. Despite these limitations, our study findings provide useful insights on the level of access to primary healthcare services and associated factors in urban slums in Nairobi, Kenya and serve as a basis for more rigorous investigations in other urban slum settings in Kenya and sub-Saharan Africa.

## Conclusions

This study shows that access to primary healthcare services for slum residents is poor and varies by the gender of the household head, source of primary care and OOP healthcare expenditure. Our study provides important results particularly highlighting the lower access to primary healthcare among female-headed households. Multiple approaches to primary healthcare access are needed to address this issue among slum residents. Therefore, policies that encourage economic growth in female-headed households are likely to create economic empowerment and improve access to primary healthcare in these settings. Similarly, public programs that offer protection against out-of-pocket spending are likely to improve access to primary healthcare. In addition, public health facilities need to improve the quality services provided.

## Data Availability

Data are available upon reasonable request. Study materials and de-identified data that support the findings in this study are available by contacting Shukri Mohamed at the African Population and Health Research Center in Kenya via email address smohamed@aphrc.org.

## Abbreviations

AOR: Adjusted odds ratio
CI: Confidence interval
KHEUS: Kenya household healthcare expenditure and utilization survey
LMIC: Low and middle-income countries
NUHDSS: Nairobi urban health demographic surveillance system
OOP: Out of pocket payment
PCA: Principal component analysis
UHC: Universal health coverage
USD: United States Dollar

## Footnotes

1 One assumption underlying the ordered logistic is the parallel lines assumption/proportional odds which posits that the relationship between each pair of the outcome groups is the same.
